# Huaier attenuates the adverse effects of pyroptosis by regulating the methylation of rat mesangial cells: an in vitro study

**DOI:** 10.1186/s12906-022-03559-4

**Published:** 2022-03-29

**Authors:** Wenjia Geng, Can Tu, Dahao Chen, Zhaoyu Lu, Wei Mao, Hanyu Zhu

**Affiliations:** 1 Peking University People’s Hospital, Beijing, China; 2 The Second Affiliated Hospital of Guangzhou University of Chinese Medicine, Guangzhou, China; 3 Department of nephrology, Wuhan Hospital of Traditional Chinese and Western Medicine, Wuhan, China; 4 State Key Laboratory of Dampness Syndrome of Chinese Medicine, Guangzhou, China; 5 Department of nephrology, Guangdong Provincial Hospital of Chinese Medicine, 111 Dade Road, Yuexiu District, Guangzhou, Guangdong province China; 6 Department of Nephrology, The First Medical Center, Chinese PLA General Hospital, Chinese PLA Institute of Nephrology, State Key Laboratory of Kidney Diseases, National Clinical Research Center for Kidney Diseases, No.28 Fuxing Road, Haidian District, Beijing, China

**Keywords:** Rat mesangial cells, Pyroptosis, Huaier, GSDME, Methylation

## Abstract

**Background:**

Pyroptosis is a highly programmed inflammatory cell death process that represents an innate immune response. In this study, the occurrence of pyroptosis in rat mesangial cells (RMCs) and the effect of Huaier (Trametes robiniophia Murr) on this process were investigated.

**Methods:**

RMCs were incubated with OX7 antibodies (0.5 μg/ml, 2.5 μg/ml, 10 μg/ml), normal rat serum (NRS) and Huaier (1 mg/ml, 5 mg/ml, 10 mg/ml). RMC morphology was observed under a light microscope and by immunofluorescence. Lactate dehydrogenase (LDH) release was assessed using the CytoTox 96 Non-Radioactive Cytotoxicity Assay Kit. Western blot assays were performed, and then the RMCs were incubated with the methylase DNMT3B and the demethylase 5-aza-2′-deoxycytidine.

**Results:**

Morphological, LDH, immunofluorescence and western blot analyses showed that RMCs were lysed when stimulated with OX7 antibodies and NRS. RMC lysis released inflammatory cytokines (interleukin-18, interleukin-1β, monocyte chemoattractant protein-1 and intracellular adhesion molecule-1), and Huaier protected RMCs by controlling lysis and the levels of inflammatory cytokines. Lysis was mediated by pyroptosis due to the positive expression of GSDME. The methylase DNMT3B reduced the expression of GSDME induced by OX7 together with NRS. Furthermore, Huaier significantly suppressed the expression of GSDME, which was increased by 5-aza-2’-deoxycytidine.

**Conclusions:**

Pyroptosis might occur in RMCs, and Huaier can protect RMCs by upregulating the methylation of a group of molecules.

**Supplementary Information:**

The online version contains supplementary material available at 10.1186/s12906-022-03559-4.

## Introduction

Pyroptosis is a highly programmed inflammatory cell death process that is an innate immune response [1, 2]. GSDME (DFNA5) is one of the main executor proteins of pyroptosis and can be cleaved by activated caspase-3. The self-inhibition of GSDME is released, and N-terminal fragments are exposed. The N-terminal fragments perforate in the cell membrane, thereby causing pyroptosis [3, 4].

Recently, an increasing number of studies have shown that the caspase-3/GSDME-dependent pyroptosis pathway plays a role in kidney disease [5–7]. However, in the field of mesangial proliferative glomerulonephritis (MsPGN), there is almost no research on pyroptosis. MsPGN is one of the most common pathological symptoms among chronic kidney diseases such as IgA nephropathy and membranous nephropathy. Acute anti-thy1 MsPGN in rats is a classic and stable model to mimic the pathogenesis of MsPGN [8]. This model is established by tail-vein injection of OX7 antibody (a monoclonal anti-Thy 1.1 antibody). This model is characterized by a decrease in the number of mesangial cells (MCs) in 1–3 days, with the least number of cells on day 3. Then MCs proliferate gradually, reaching the highest cell number at 7 days and then gradually decreasing. Previously, it was thought that this cellular reduction was caused by C5b-9 attacking mesangial cells to induce apoptosis [9]. Then, it was discovered that attack may initiate signal transduction leading to apoptosis and DNA rupture in the nucleus [10]. This phenomenon is similar to pyroptosis. Therefore, this study simulated the model of acute anti-thy1 MsPGN in vitro.

Urine protein excretion and the proliferation of MCs in acute anti-Thy-1 MsPGN might be inhibited by Trametes robiniophia Murr (Huaier), as we previously reported [11]. As a medicinal fungus in China, Huaier was first described by Gehong in the 340 s. Another traditional Chinese medicine book stated that Huaier dispels wind, breaks blood and reinforces a healthy qi. A series of pharmacological studies showed that Huaier could promote tumor cell apoptosis and inhibit tumor cell proliferation [12, 13], increase sensitivity to chemotherapy drugs [14], promote autophagy [15, 16] and regulate the immune response [13, 17]. In kidney disease, Huaier protects podocytes against adriamycin-induced cytotoxicity in the context of nephropathy, possibly by reversing the mitochondrial dysfunction via PGC-1α overexpression [18]. However, whether Huaier interferes with pyroptosis is unknown. Therefore, we investigated whether pyroptosis occurred in RMCs and whether Huaier played a renoprotective role by affecting pyroptosis and also assessed the mechanism by which Huaier exerts its renal protective effect. We hope to provide new ideas for a better understanding of human MsPGN pathogenesis in the future and new therapeutic drugs for the treatment of MsPGN from the perspective of traditional Chinese medicine.

## Methods

### Materials

The RMC (HBZY-1) line was obtained from the American Type Culture Collection (ATCC, Manassas, VA, USA) and incubated at 37 °C in an atmosphere of 5% CO_2_/95% O_2_. The monoclonal OX7 antibody was kindly provided by the People’s Liberation Army General Hospital. Normal rat serum (NRS) was prepared from the sera of 10 male Sprague-Dawley (180–220 g, 6–8 weeks) rats and filtered with a 0.22 μm sterile filter. All SD rats were provided by the Laboratory Animal Services Center of Guangzhou University of Chinese Medicine (Guangzhou, China). The rats were housed in the specific-pathogen-free (SPF) animal breeding room of the Academy of Chinese Medicine of Guangdong Province and given free access to water. After 1 week of adaptive feeding, the rats were anaesthetized, and blood was collected. All procedures and assays were approved by the Ethics Committee of Animal Experiments, Guangdong Provincial Hospital of Chinese Medicine (approval no. 2019041). All the protocols were performed in accordance with relevant guidelines and regulations. The study is also in accordance with the ARRIVE guidelines.

Huaier extract is the electuary ointment of Huaier (provided by Gaitianli Medicine, Jiangsu, China), which is obtained by extracting Huaier fungi by using an aqueous solution, ultrafiltration and concentration [19]. In brief, Huaier fungi were heated and boiled in an aqueous solution for 6 h. Then, the supernatant was collected, and the residue was boiled with the aqueous solution again. This process was repeated 3 times. The collected supernatants were combined, centrifuged, and filtered with a 1–30 kDa ultrafiltration membrane. Then, the supernatants were vacuum dried under reduced pressure to obtain the Huaier extract.

One gram of Huaier extract was dissolved in 10 ml of phosphate-buffered saline (PBS). Then, the Huaier solution was filtered with a 0.22 μm sterile filter and stored at − 20 °C.

### Morphology of pyroptotic cells

In this study, OX7 plus NRS was used for modeling. OX7 is an anti-thy 1 monoclonal antibody that can specifically bind to the thy-1 antigen on RMCs to form an antigen-antibody complex. NRS contain complement so that using OX7 plus NRS can simulate the pathogenesis of acute anti-thy1 MsPGN in vivo [20, 21]. RMCs at passages 5 to 10 were grown in RPMI media (Gibco) with 10% fetal bovine serum (FBS). Then, the cells were placed in 6-well plates at 4.8 × 10^5^ cells/well and allowed to adhere overnight. After being washed three times with PBS, the cells were placed in serum-free media for an additional 12 h. The cells were sensitized by incubation with OX7 antibodies (0.5 μg/ml, 2.5 μg/ml and 10 μg/ml) at 37 °C for 60 min, followed by incubation with 8% NRS or serum-free RPMI. Moreover, Huaier (10 mg/ml) or an equal volume of PBS was added. RMC morphology was observed and recorded by inverted microscopy (Olympus) within 24 h.

### Measuring cell lysis

RMCs (4 × 10^3^/ml) were cultured in 96-well plates and incubated with OX7 (2.5 μg/ml) at 37 °C for 60 min. Then, the cells were then incubated with 8% NRS or serum-free RPMI containing different Huaier concentrations (1 mg/ml, 5 mg/ml, 10 mg/ml) or an equal volume of PBS. At 2 h, 4 h and 8 h, 50 μl of supernatant was placed in a new 96-well plate after centrifugation. Then, 50 μl of CytoTox 96 Reagent was added to each well, and the plate was covered with foil to protect it from light according to the instructions of the CytoTox 96 Non-Radioactive Cytotoxicity Assay Kit (Promega Corporation). The 96-well plates were incubated for 30 min at room temperature. Finally, 50 μl of stop solution was added to each well, and the absorbance at 490 nm was analyzed.

### Immunofluorescence analysis of GSDME and OX7 in RMCs

RMCs (1.2 × 10^5^/ml) were seeded in 6-well plates and grown to 30–40% confluence. Cells were incubated with OX7 (2.5 μg/ml) at 37 °C for 60 min and then incubated with 8% NRS or serum-free RPMI. Different Huaier solutions (1 mg/ml, 5 mg/ml, 10 mg/ml) were added concurrently. After being incubated for 4 h, the culture medium was removed. Then, the cells were washed with PBS three times, fixed with 4% paraformaldehyde, permeabilized with 0.5% Triton X-100 at 4 °C and subsequently blocked with 0.1% bovine serum albumin (BSA). Thereafter, the cells were incubated with anti-GSDME antibodies (55 kD, 35 kD, 25 kD, sc393162, Santa Cruz) or OX7 antibodies at 4 °C overnight. On the second day, Cy3-labeled goat anti-mouse antibodies were added and incubated for 1 h at room temperature. DAPI was added to stain the nuclei. Then, the expression of GSDME or OX7 in RMCs was observed by laser scanning confocal microscopy. In addition, it should be noted that GSDME 55 kD is GSDME full length (GSDME-FL), GSDME 35 kD is the GSDME N terminus (GSDME-NT), and GSDME 25 kD is the GSDME C terminus (GSDME-CT) [4, 22]. After activation of GSDME-FL, GSDME-NT and GSDME-CT can be formed. The perforating effect that causes pyroptosis is mainly accomplished by GSDME-NT [23].

### Western blot analysis of the expression of GSDME and related proteins in RMCs

RMCs (4.8 × 10^5^/ml) were seeded in 6-well plates and grown to 50% confluence. Cells were incubated with OX7 (2.5 μg/ml) at 37 °C for 60 min. Then, the cells were incubated with 8% NRS or serum-free RPMI and different Huaier concentrations (1 mg/ml, 5 mg/ml, 10 mg/ml) or an equal volume of PBS. After being incubated for 4 h, the culture medium was removed. The cells were washed with PBS three times. After removing the media, the cells were lysed with RIPA buffer (Thermo Fisher Scientific, USA) and collected in 1.5 ml EP tubes. Approximately 20 μg of protein was loaded into each lane, and after electrophoresis the proteins were transferred onto PVDF membranes. The primary antibodies included anti-mouse monoclonal GSDME, anti-rabbit polyclonal PARP (116 kD, #9542, CST), anti-polyclonal caspase-3 (35 kD, 19 kD, 17 kD, #9662, CST), monoclonal rabbit IL-18 (44 kD, 52,914, Abcam), and polyclonal anti-MCP-1 (13–15 kD, NBP1–07035, Novusbio). The membranes were then incubated with secondary anti-mouse IgG (#7076, CST) labelled with HRP or anti-rabbit IgG (#7074, CST) labelled with HRP. The levels of GAPDH, detected by a rabbit monoclonal antibody (37 kD, #2118, CST), β-actin, detected by an anti-monoclonal mouse antibody (43 kD, #3700, CST) and αβ-tubulin, detected by an anti-polyclonal rabbit antibody (55 kD, #2148, CST) were measured as loading controls. Immunostained bands were detected using an ECL kit (Bio-Rad, California, USA).

### GSDME expression was reduced by DNMT3B

RMCs (4.8 × 10^5^/ml) were seeded in 6-well plates and grown to 50% confluence. Cells were incubated with OX7 (2.5 μg/ml) at 37 °C for 60 min and then incubated with 8% NRS or serum-free RPMI. Furthermore, DNMT3B (1 nmol, 10 nmol, 20 nmol, 40 nmol, 400 nmol) was added, and serum-free RPMI was added to the control group. After 4 h, cell culture was terminated, protein was extracted from the after two washes with PBS, and the expression of GSDME was determined.

### GSDME expression was increased by 5-aza-DC and decreased by Huaier

RMCs (1.2 × 10^5^/ml) were seeded in 6-well plates. Cells were incubated with OX7 (2.5 μg/ml) at 37 °C for 60 min and then incubated with 8% NRS or serum-free RPMI. Moreover, Huaier (1 mg/ml), 5-aza-DC (10 μM) or an equal volume of PBS was added. After 48 h of incubation, the culture medium was removed. Then, the cells were washed with PBS three times. Cellular proteins were extracted, and we performed western blot analysis as described above.

### Statistical analysis

The data were analyzed by SPSS 23.0 (New York, USA) and GraphPad Prism 5.01 (San Diego, USA). For continuous variables, analysis of variance was used for normally distributed data, and the Kruskal-Wallis H test was used for nonnormally distributed data. Pairwise comparisons were performed using the least significant difference *t* test. *P* < 0.05 was considered statistically significant.

## Results

### OX7 antibodies plus NRS induced RMC lysis, while Huaier protected RMCs

**Fig. 1:**
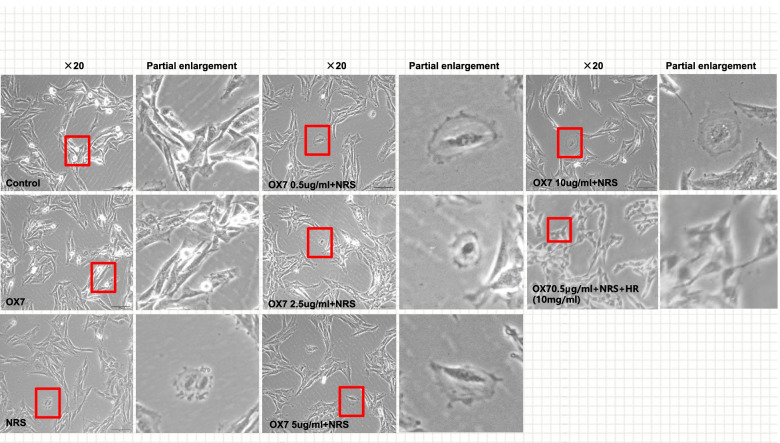
Morphological changes in RMCs after OX7 (0.25 μg/ml,2.5 μg/ml,5 μg/ml,10 μg/ml), 8% NRS and Huaier (10 mg/ml) stimulation under a light microscope

**Fig. 2:**
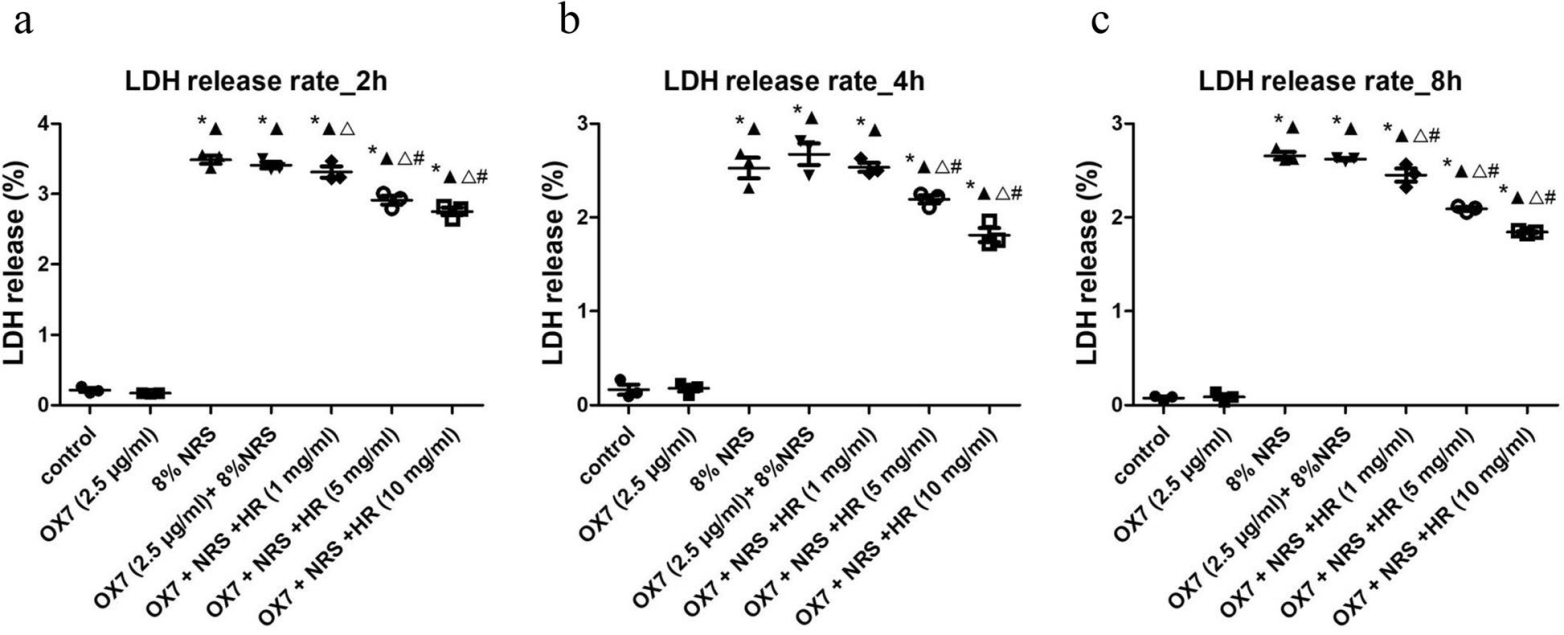
LDH release from RMCs after OX7, NRS and Huaier stimulation (2a, LDH release in each group at 2 h; 2b, LDH release in each group at 4 h; 2c, LDH release in each group at 6 h). *, compared to the control group, *P* < 0.05. #, compared to the OX7 + NRS group, *P* < 0.05. ▲, compared to the OX7 group, *P* < 0.05. △, compared to the NRS group, *P* < 0.05. #, compared to the OX7 + NRS group, *P* < 0.05

**Fig. 3:**
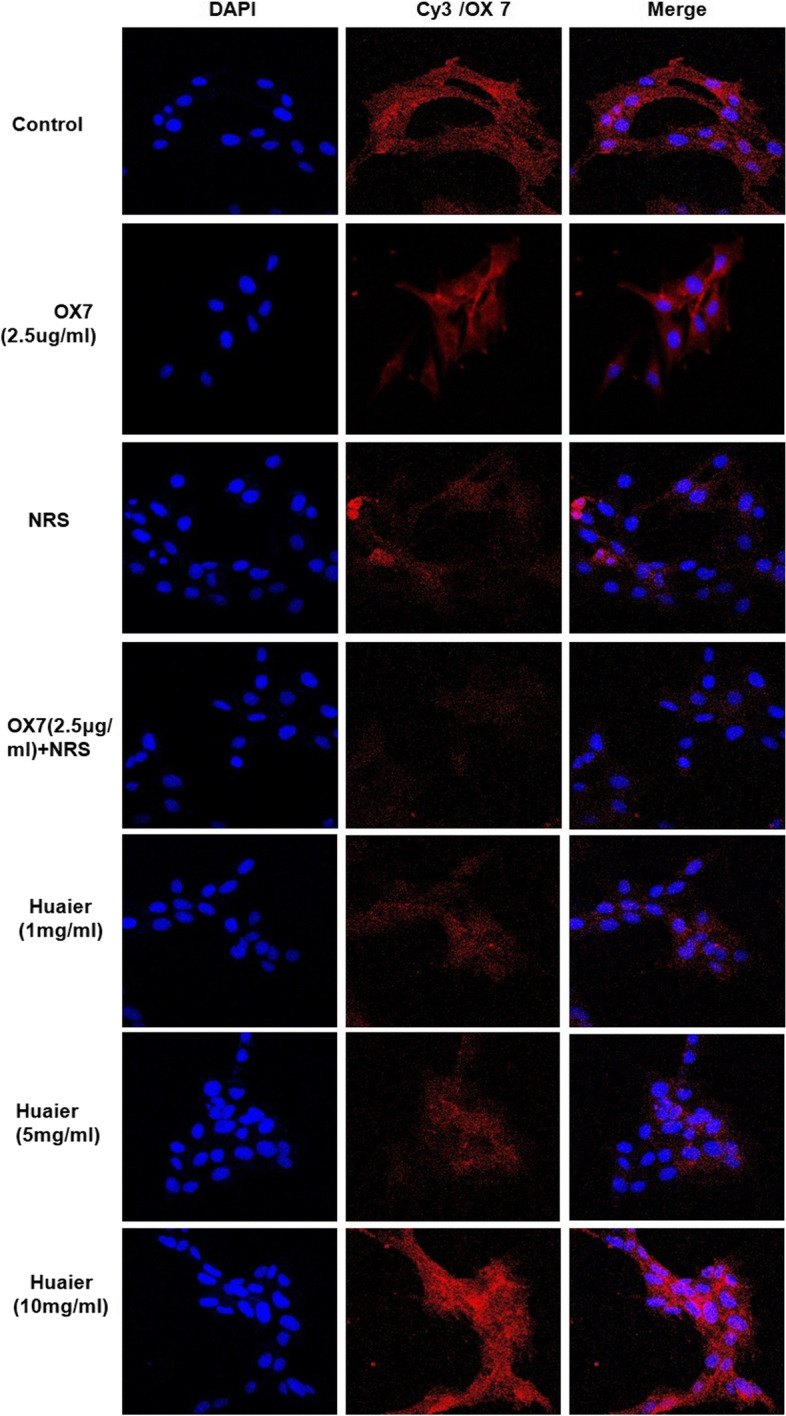
Morphological changes in RMCs after OX7, NRS and Huaier stimulation, as shown by immunofluorescence analysis

First, RMCs in the control and the OX7 group grew normally or underwent apoptosis, as shown by microscopy. In contrast, cells stimulated with OX7 (0.5 μg/ml, 2.5 μg/ml, 10 μg/ml) plus NRS experienced membrane swelling and subsequent lysis, resulting in the release of cytosolic contents. Moreover, RMCs in the Huaier (10 mg/ml) group grew normally (Fig. 1). Second, compared with the OX7 + NRS, Huaier (5 mg/ml and 10 mg/ml) significantly reduced LDH release at 2 h and 4 h. At 8 h, compared with the OX7 + NRS group (Fig. 2a, b and c). Third, RMCs in the control group appeared to have obvious membranes, while cells in the OX7 + NRS group showed a weakened membrane, as indicated by immunofluorescence. Control and OX7 + NRS RMCs had intact nuclei. Moreover, Huaier (5 mg/ml) partially protected RMCs, and Huaier (10 mg/ml) protected RMCs even better than the lower dose (Fig. 3).

**Fig. 4:**
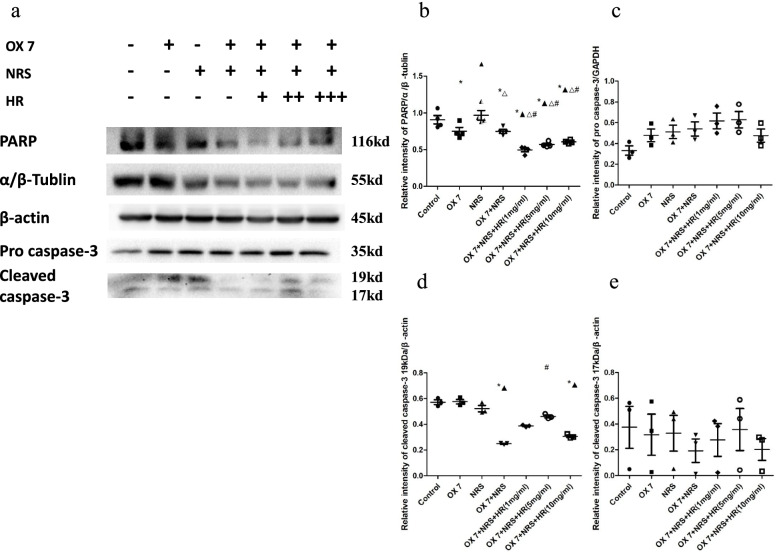
Expression of caspase-3 and PARP in RMCs stimulated by OX7, NRS and Huaier. (**a** western blot results of pro-caspase-3, cleaved caspase-3 and PARP; **b** relative intensity ratio of caspase-3 to β-actin; **c** relative intensity ratio of caspase-3 to α/β-tubulin; **d** relative intensity ratio of cleaved caspase-3 19 kD to β-actin; **e** relative intensity ratio of cleaved caspase-3 17 kD to β-actin). +, Huaier 1 mg/ml; ++, Huaier 5 mg/ml; +++, Huaier 10 mg/ml. *, compared to the control group, *P* < 0.05. #, compared to the OX7 + NRS group, *P* < 0.05. ▲, compared to the OX7 group, *P* < 0.05. △, compared to the NRS group, *P* < 0.05. #, compared to the OX7 + NRS group, *P* < 0.05

Cleaved caspase-3 expression in the OX7 + NRS group was decreased compared to that in the control group. However, Huaier increased the expression of cleaved caspase-3 (Fig. 4a, d and e). There was no differences in pro caspase-3 (Fig. 4a and b). In addition, poly ADP-ribose polymerase (PARP) expression in the OX7 + NRS group was significantly decreased compared to that in the control, OX7 and NRS groups. However, Huaier increased PARP expression in a concentration-dependent manner (Fig. 4a and c). The low expression levels of cleaved caspase-3 and PARP in the OX7 + NRS group indicated the consumption or inactivation of these factors, which needs to be further verified.

### RMC lysis released inflammatory cytokines, which was reduced by Huaier

**Fig. 5:**
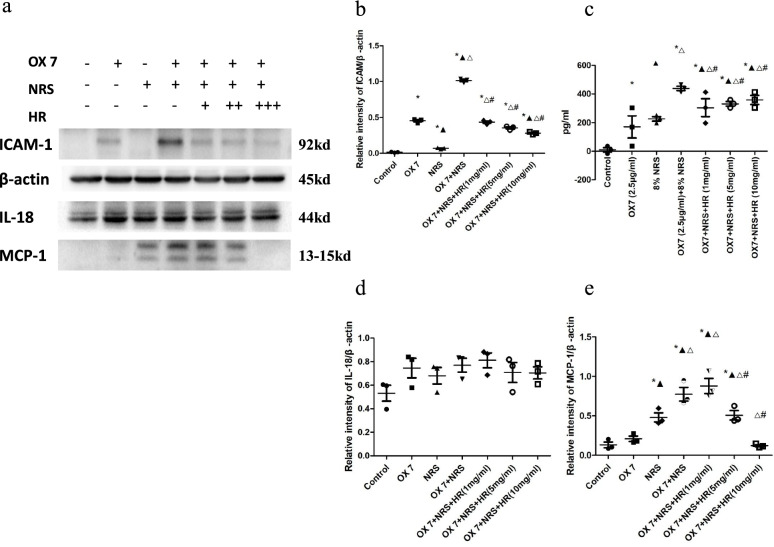
Expression of IL-18, MCP-1, ICAM-1 and IL-1β after RMCs were stimulated by OX7, NRS and Huaier (**a** relative intensity ratio of ICAM to β-actin; **b** expression of IL-1β in the cell culture supernatant; **c** relative intensity ratio of IL-18 to β-actin; **d** relative intensity ratio of MCP-1 to β-actin). +, Huaier 1 mg/ml; ++, Huaier 5 mg/ml; +++, Huaier 10 mg/ml. *, compared to the control group, *P* < 0.05. #, compared to the OX7 + NRS group, *P* < 0.05. ▲, compared to the OX7 group, *P* < 0.05. △, compared to the NRS group, *P* < 0.05. #, compared to the OX7 + NRS group, *P* < 0.05

IL-18, MCP-1, ICAM-1 and IL-1β were significantly increased compared to those in the control group. However, in the Huaier (10 mg/ml) group, there were decreased in the expression levels of ICAM-1, IL-18 and MCP-1 (Fig. 5a, b, d and e). Although Huaier also decreased the expression of IL-1β, there was no significant difference (Fig. 5c).

### RMC lysis was mediated by pyroptosis, and Huaier reduced the expression of GSDME

**Fig. 6:**
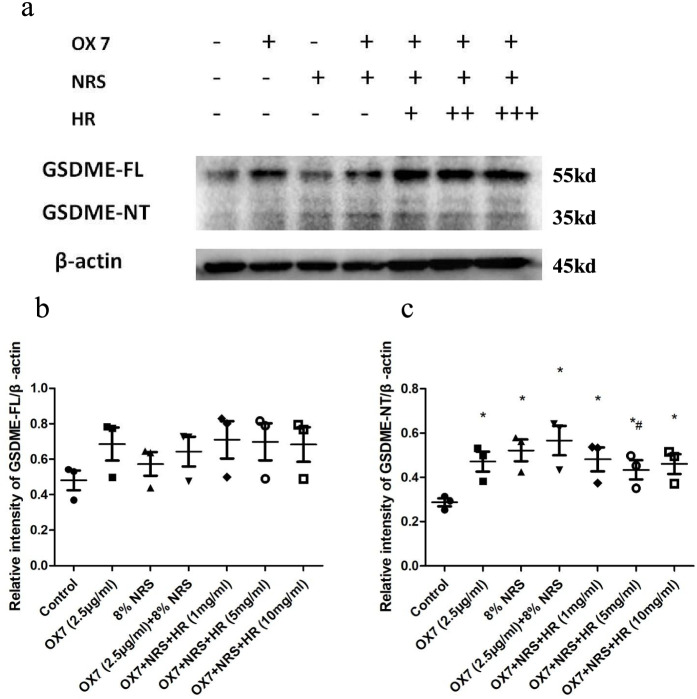
Expression of GSDME after RMCs were stimulated with OX7, NRS and Huaier, as shown by western blotting (**a** western blot results of GSDME-FL and GSDME-NT; **b** relative intensity ratio of GSDME-FL to β-actin; **c** relative intensity ratio of GSDME-NT to β-actin). +, Huaier 1 mg/ml; ++, Huaier 5 mg/ml; +++, Huaier 10 mg/ml. *, compared to the control group, *P* < 0.05. #, compared to the OX7 + NRS group, *P* < 0.05. #, compared to the OX7 + NRS group, *P* < 0.05

**Fig. 7:**
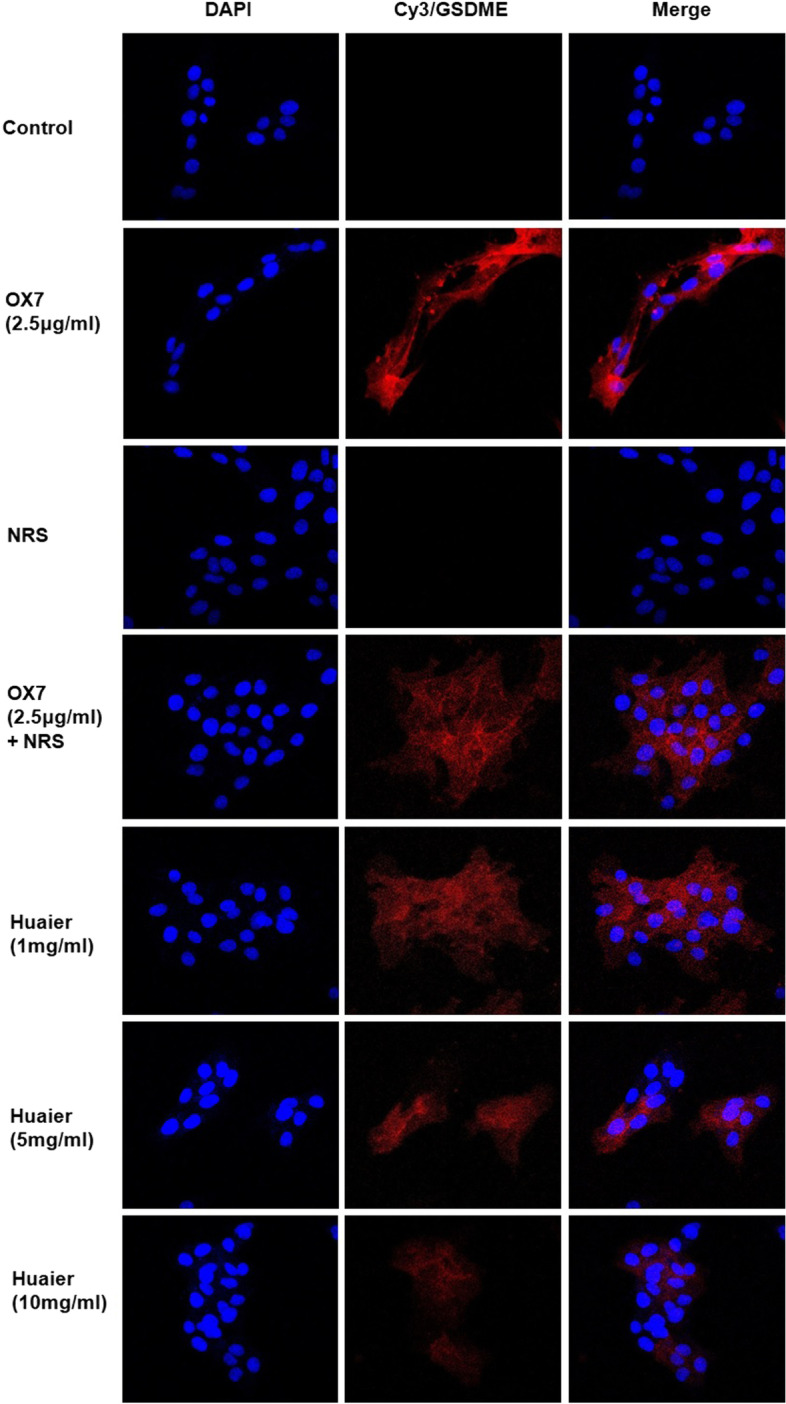
Expression of GSDME after RMCs were stimulated by OX7, NRS and Huaier, as shown by immunofluorescence analysis

According to Shaofeng’s research [4], GSDME is a new biomarker of pyroptosis in many cell types. GSDME-NT is the fragment that forms holes in the cell membrane and induces pyroptosis. Hence, we examined the expression of GSDME by western blotting and immunofluorescence assays. We found that the expression of GSDME in the control group and the NRS group was low, while GSDME-NT levels in the OX7 group and the OX7 + NRS group increased notable, as shown by western blotting. And the expression of GSDME was significantly reduced in Huaier (5 mg/ml) group than in the OX7 + NRS group (Fig. 6a, b and c). Consistent with this result, immunofluorescence analysis of GSDME showed that the control group was negative, while the OX7 group and the OX7 + NRS group were positive. The positive signal was attenuated by increasing concentrations of Huaier (Fig. 7).

### The initiating methylase DNMT3B could reduce RMC pyroptosis induced by OX7 antibodies plus complement

**Fig. 8:**
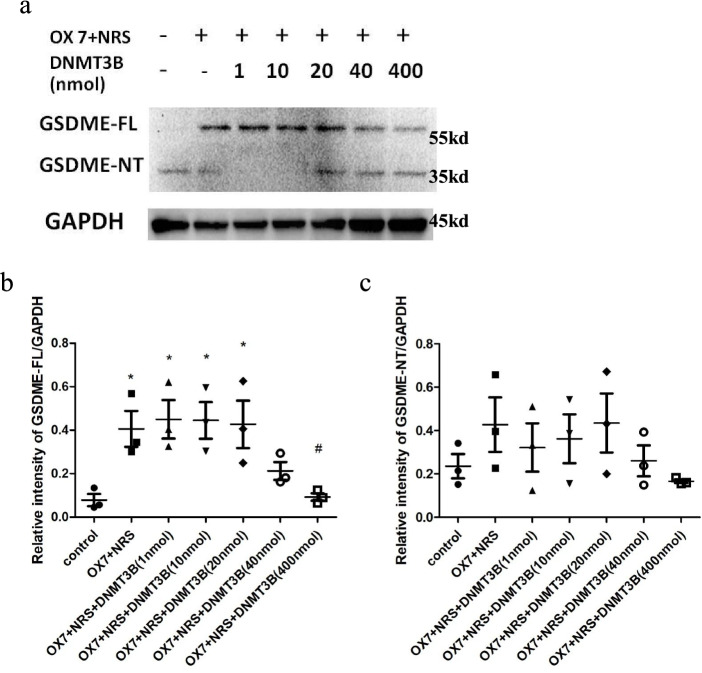
The methylase DNMT3B can attenuate GSDME expression in RMCs induced by OX7 plus NRS (**a** western blot results of GSDME-FL and GSDME-NT; **b** relative intensity ratio of GSDME-FL to GAPDH; **c** relative intensity ratio of GSDME-NT to GAPDH). +, OX7 (0.5 μg/ml) plus Huaier (1 mg/ml). *, compared to the control group, *P* < 0.05. #, compared to the OX7 + NRS group, *P* < 0.05. #, compared to the OX7 + NRS group, *P* < 0.05

DNMT3B is an initiating DNA methyltransferase that catalyzes the formation of methylation sites on unmethylated DNA double strands and downregulates gene expression. Compared with the control group, the OX7 + NRS, OX7 + NRS + DNMT3B (1 nmol), OX7 + NRS + DNMT3B (10 nmol), and OX7 + NRS + DNMT3B (20 nmol) groups exhibited significant differences. Likewise, there was a significant reduction in the OX7 + NRS + DNMT3B (400 nmol) group compared with the OX7 + NRS group (Fig. 8a, b and c).

### 5-Aza-2’-deoxycytidine (5-Aza-DC) increased GSDME expression induced by OX7 antibodies plus NRS, while Huaier attenuated this expression

**Fig. 9:**
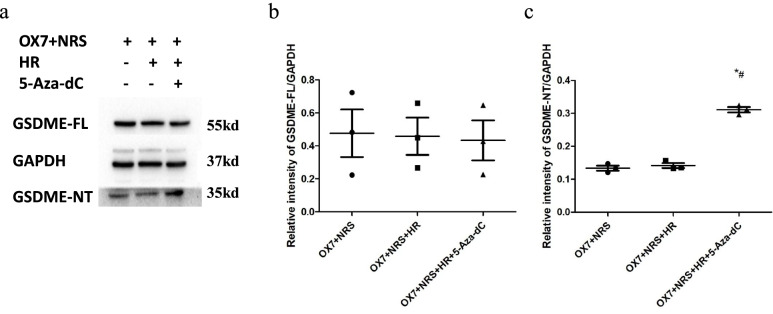
The demethylating agent 5-aza-DC can upregulate the expression of GSDME in RMCs, while Huaier can attenuate the upregulated GSDME expression (**a** western blot results of GSDME-FL and GSDME-NT; **b** relative intensity ratio of GSDME-FL to GAPDH; **c** relative intensity ratio of GSDME-NT to GAPDH). +, OX7 (0.5 μg/ml) plus Huaier (1 mg/ml).*, compared to the OX7 + NRS group, *P* < 0.05. #, compared to the OX7 + NRS + HR group, *P* < 0.05

In contrast to DNMT3B, 5-aza-DC causes the demethylation of DNA. In cancer cell lines, this compound can reverse the effect of silencing GSDME [24]. In this study, 5-aza-DC increased the expression of GSDME after the cells were incubated with OX7 and NRS. Huaier is known as Trametes robiniophila Murr and grows on the stalks of old locust trees. The results of the present study showed that Huaier could reduce the 5-aza-DC-enhanced expression of GSDME. These results suggested that Huaier could exert an antipyroptotic effect by enhancing GSDME methylation (Fig. 9a, b and c).

## Discussion

In this study, we found that RMC pyroptosis could be induced by OX7 antibodies plus NRS, and Huaier could play a renoprotective role by affecting pyroptosis. Mesangial cells can undergo pyroptosis consistent with previous studies. Yuxuan Zhang et al. [25] found that mesangial cells undergo pyroptosis in rats with unilateral ureteral obstruction (UUO). Jin-Feng Zhan et al. [26] found that lncRNA-Neat1 can promote high glucose-induced rat MC pyroptosis.

Huaier can play a protective role by suppressing pyroptosis and the release of inflammatory factors in MCs. Huaier is the fruiting body of Trametes robiniophila Murr, which grows on the rods of aged *Sophora japonica* trees and belongs to the Basidiomycota phylum, the Polyporaceae family, and the Trametes genus [27]. Huaier was first published in Behind the Elbow and can cure colonic wind poison. Previous studies have confirmed that Huaier can play an antitumor role by regulating immune functions, such as increasing the index of immune organs, regulating the number of lymphocytes and regulating the secretion of cytokines. Ying Chen et al [28] found that Huaier could increase the expression of Duffy antigen receptor for chemokines (DARC) and reduce its ligands, such as CC chemokine ligand 2 (CCL-2), CXC chemokine ligand 8 (CXCL-8, IL-8), matrix metalloproteinase 2 (MMP-2), and CXC chemokine ligand 1 (CXCL-1). DARC plays a negative role in breast cancer metastasis. Yi Sun et al. [13] found that Huaier polysaccharide can inhibit the proliferation of cholangiocarcinoma on the one hand and promote the proliferation of spleen cells in BALB/c mice on the other hand, inducing the production of nitric oxide synthase and thus making macrophages produce more nitric oxide and enhancing macrophage phagocytosis. These studies confirmed the immunomodulatory role of Huaier.

However, our findings are contrary to those of Jun Xie et al., who found that Huaier increased the pyroptosis of non-small-cell lung cancer cells, thereby reducing their proliferation and playing a therapeutic role [29]. Tumor cells were the object of Jun Xie’s study, which showed that increasing pyroptosis reduced proliferation. This is consistent with the findings of Feng Shao. However, MCs are a normal cell type inherent in the kidney. In addition to being a form of cell death, pyroptosis can cause a strong inflammatory response [30]. In recent years, pyroptosis has been gradually characterized by swelling, rupture, and the loss of intact cell morphology [31]. Pyroptotic cells release their cellular contents, causing a strong inflammatory response and ultimately activating the innate immune response. Excessive inflammatory responses might also stimulate the excessive proliferation of normal cells [32]. Our research results suggest that Huaier can suppress the pyroptosis of RMCs and the release of inflammatory factors, which may attenuate the excessive proliferation of cells caused by the inflammatory response, thereby exerting a protective effect on renal cells.

Further, Huaier could play a protective role by upregulating the methylation of a group of molecules in MCs. GSDME is silenced due to promoter methylation in cancers [24, 33, 34]. DNA methylation is catalyzed by DNA methyltransferases (DNMTs), which transfer a methyl group to the 5th carbon atom of cytosine to form 5-methylcytosine (5MC). DNMT3A and DNMT3B can establish new methylation patterns on unmodified DNA, and these are called initiating methylation enzymes, while DNMT1 copies DNA methylation patterns from parent DNA strands to newly synthesized daughter DNA strands during DNA replication. This study confirmed that the expression of GSDME could be attenuated by DNMT3B, which was consistent with Duan Y et al.’s study [35]. On the other hand, 5-aza-DC, a methyltransferase inhibitor, simulates cytosine and blocks methyltransferase activity to reduce DNA methylation levels. As an epigenetic regulatory reagent, 5-aza-DC is widely used to examine the hypomethylation of cellular DNA. Kim used 5-Aza-DC to activate silenced genes in colon cancer and found that GSDME was activated the most frequently, in up to 40% of cases [24]. The results of this study also showed that 5-Aza-DC could further increase the expression of GSDME in RMCs.

The results of our study indicated that Huaier protected RMCs by reducing pyroptosis and preventing the release of inflammatory cytokines. Pyroptosis can be activated by innate immune-dependent pattern recognition receptors (PRRs) to disrupt extraneous antigen replication and kill bacteria through perforation, which is a part of innate immunity [36, 37]. This finding suggests that Huaier-mediated regulation may also occur through the immune response. In addition, it was further shown that Huaier could reduce the upregulated GSDME expression in RMCs after demethylation, suggesting that Huaier could play a protective role by regulating RMC methylation.

## Conclusion

We found that OX7 antibodies together with NRS induce pyroptosis in RMCs. Huaier plays a protective role by upregulating the methylation of a group of molecules in RMCs.

## Supplementary Information

**Additional file 1.** : Expression of caspase-3 and PARP in RMCs stimulated with OX7, NRS and Huaier.

**Additional file 2.** : Expression of IL-18, MCP-1, ICAM-1 and IL-1β after the stimulation of RMCs with OX7, NRS and Huaier.

**Additional file 3.** : Expression of GSDME after the stimulation of RMCs with OX7, NRS and Huaier.

**Additional file 4.** : shows that the methylase DNMT3B can attenuate GSDME expression in RMCs induced by OX7 plus NRS.

**Additional file 5.** : shows that the demethylating agent 5-aza-DC can upregulate the expression of GSDME in RMCs, while Huaier can attenuate the upregulated GSDME expression.

## Data Availability

The datasets used and/or analyzed during the current study are available from the corresponding author upon reasonable request.

## Abbreviations

RMCs: Rat mesangial cells
NRS: Normal rat serum
LDH: Lactate dehydrogenase
IL-18: Interleukin-18
IL-1β: Interleukin-1β
MCP-1: Monocyte chemoattractant protein-1
ICAM-1: Intracellular adhesion molecule-1
MsPGN: Mesangial proliferative glomerulonephritis
FBS: Fetal bovine serum
PARP: Poly ADP-ribose polymerase
DNMTs: DNA methyltransferases
5-MC: 5-methylcytosine
5-Aza-DC: 5-Aza-2’-deoxycytidine
DARC: Duffy antigen receptor for chemokines
CCL-2: CC chemokine ligand 2
CXCL-1: CXC chemokine ligand 1
CXCL-8 (IL-8): CXC chemokine ligand 8
MMP-2: Matrix metalloproteinase 2
