# Supplementary figures and images for: Huaier attenuates the adverse effects of pyroptosis by regulating the methylation of rat mesangial cells: an in vitro study
Source: BMC Complement Med Ther. 2022 Mar 29;22:92. doi: 10.1186/s12906-022-03559-4 (PMC8966145; doi:10.1186/s12906-022-03559-4)

## Supplementary Information file 1

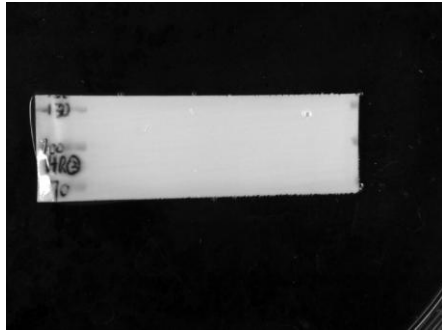

PARP

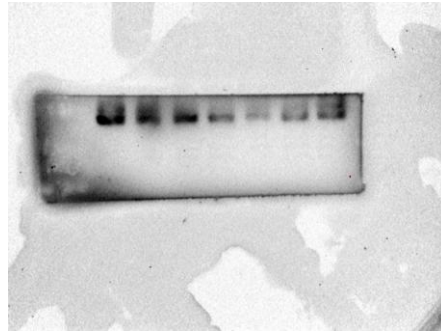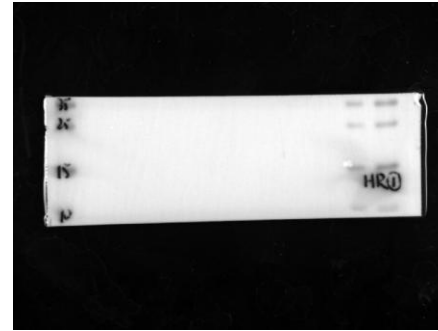

Pro caspase-3

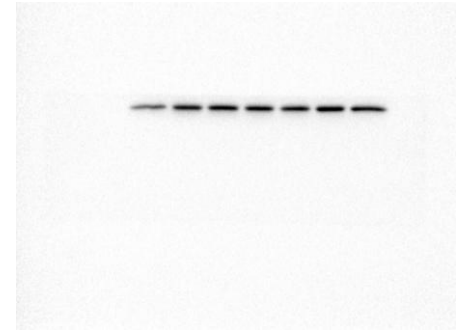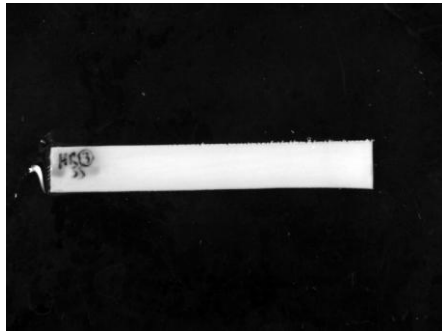

$\alpha/\beta$ -tublin

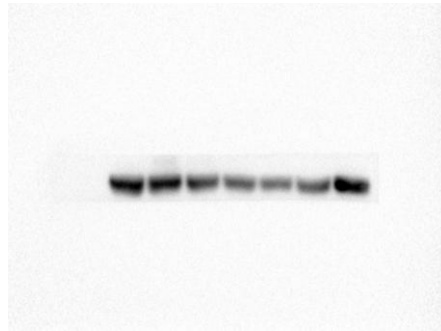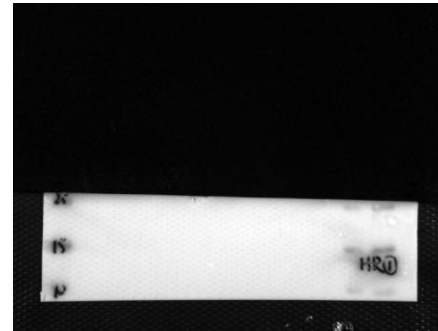

Cleaved caspase-3

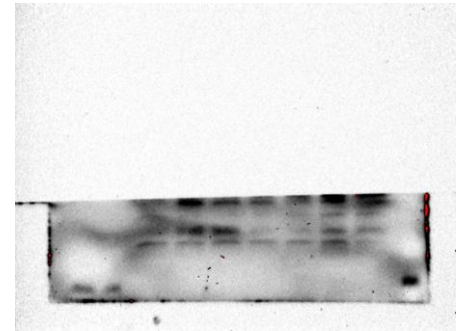

Supplement: Supplementary file 1 — Additional file 1. : Expression of caspase-3 and PARP in RMCs stimulated with OX7, NRS and Huaier. [file 12906_2022_3559_MOESM1_ESM.pdf]

## Supplementary Information file 2

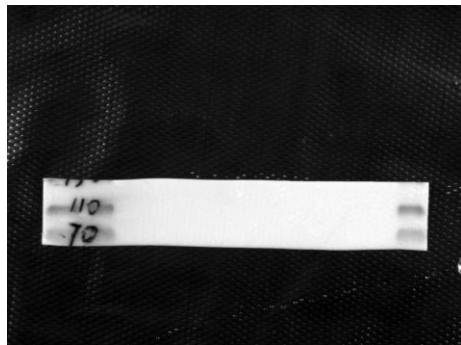

ICAM

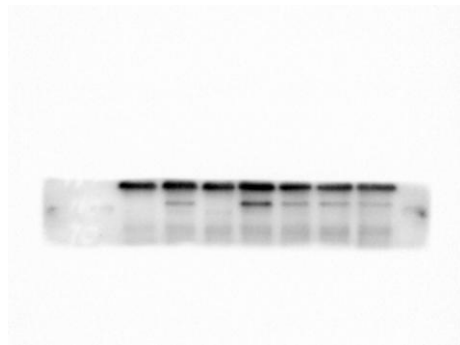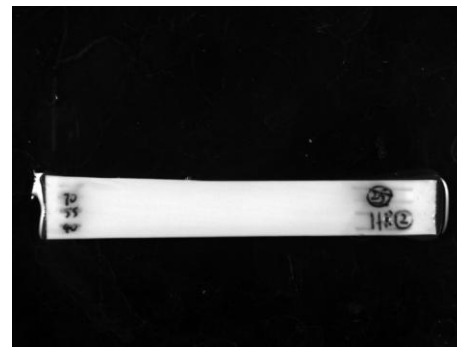

IL-18

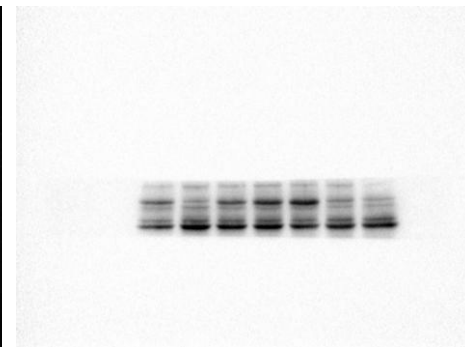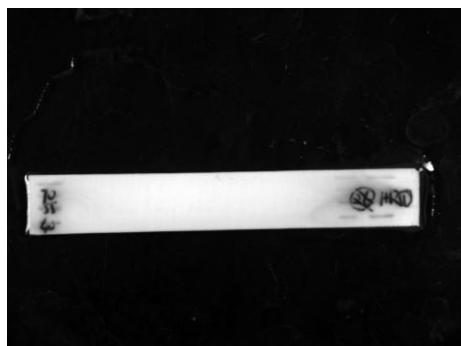

$\beta$ -actin

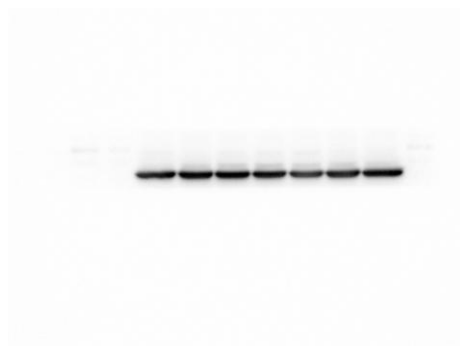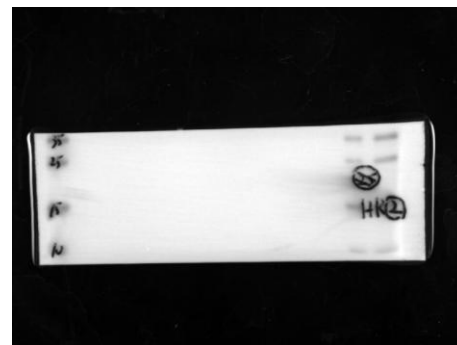

MCP-1

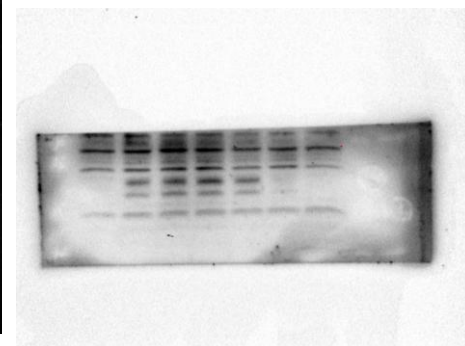

Supplement: Supplementary file 2 — Additional file 2. : Expression of IL-18, MCP-1, ICAM-1 and IL-1β after the stimulation of RMCs with OX7, NRS and Huaier. [file 12906_2022_3559_MOESM2_ESM.pdf]

## Supplementary Information file 3

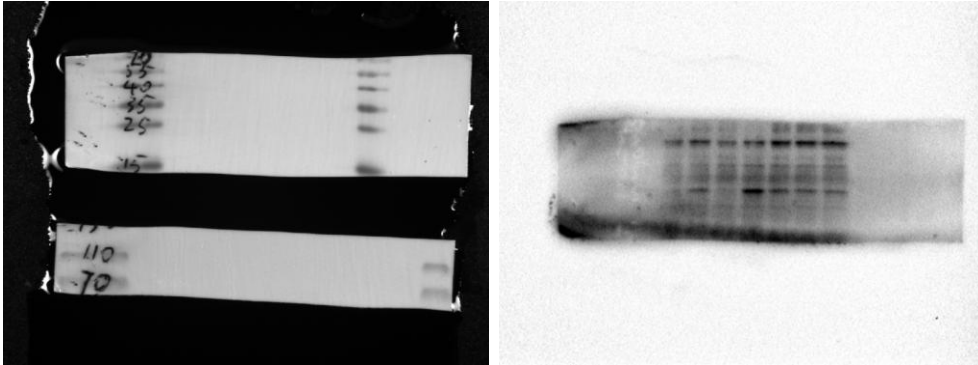

**GSDME**

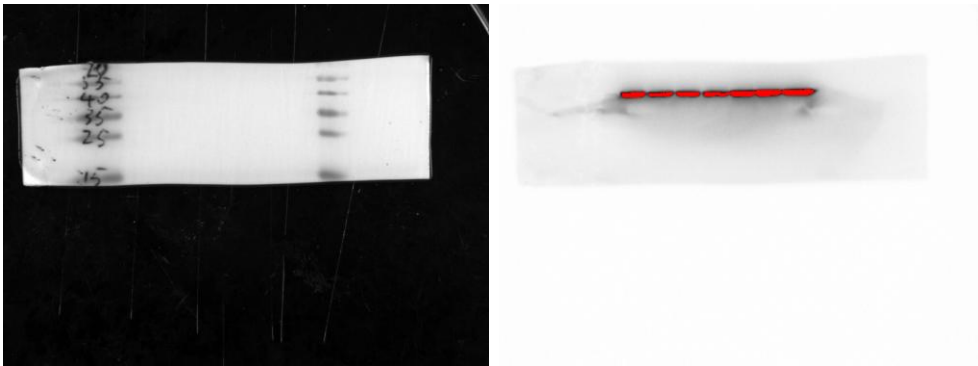

**β-actin**

Supplement: Supplementary file 3 — Additional file 3. : Expression of GSDME after the stimulation of RMCs with OX7, NRS and Huaier. [file 12906_2022_3559_MOESM3_ESM.pdf]

## Supplementary Information file 4

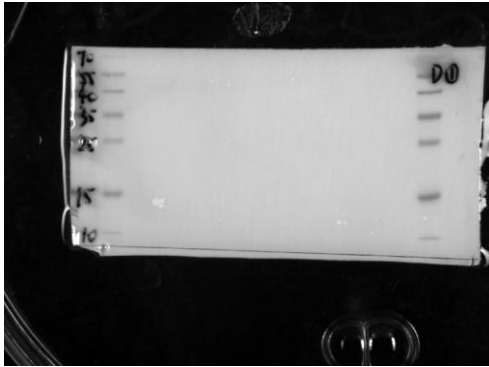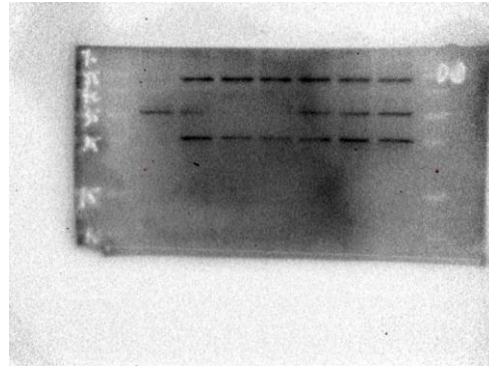

**GSDME-FL+NT**

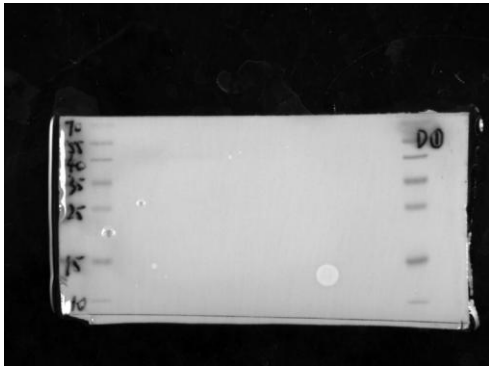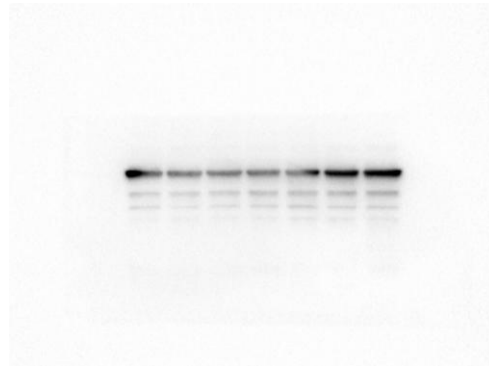

**GAPDH**

Supplement: Supplementary file 4 — Additional file 4. : shows that the methylase DNMT3B can attenuate GSDME expression in RMCs induced by OX7 plus NRS. [file 12906_2022_3559_MOESM4_ESM.pdf]

## Supplementary Information file 5

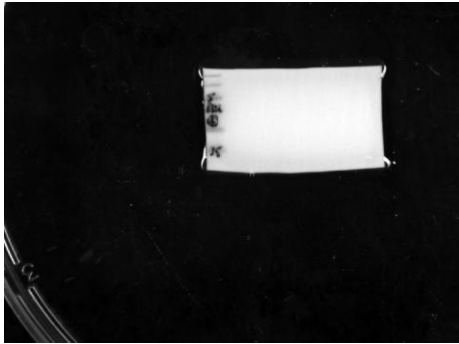

**GSDME-FL**

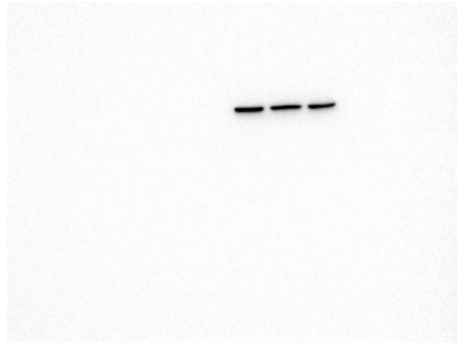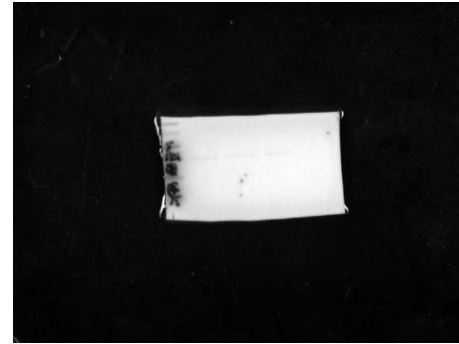

**GAPDH**

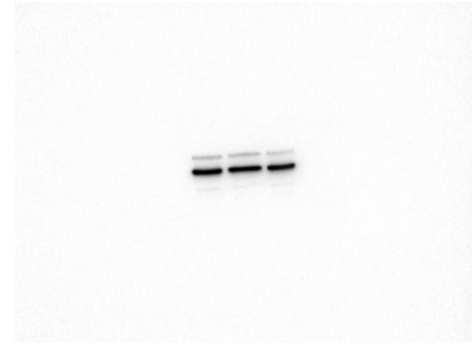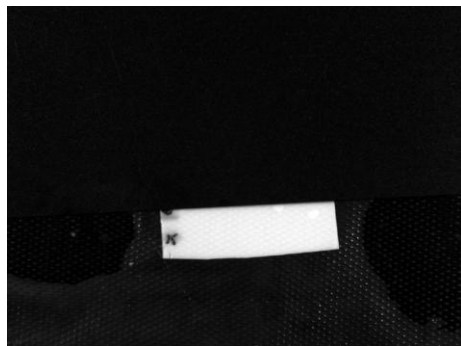

**GSDME-NT**

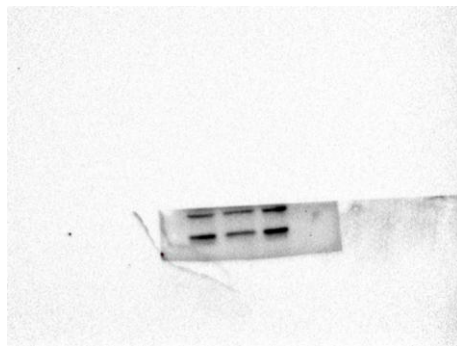

Supplement: Supplementary file 5 — Additional file 5. : shows that the demethylating agent 5-aza-DC can upregulate the expression of GSDME in RMCs, while Huaier can attenuate the upregulated GSDME expression. [file 12906_2022_3559_MOESM5_ESM.pdf]
